# Preoperative pelvic floor muscle exercise for continence after radical prostatectomy: a systematic review and meta-analysis

**DOI:** 10.3389/fpubh.2023.1186067

**Published:** 2023-07-31

**Authors:** Luqiang Zhou, Yu Chen, Xiaojuan Yuan, Lijing Zeng, Jingzhen Zhu, Ji Zheng

**Affiliations:** Department of Urologic Surgery Center, Second Affiliated Hospital, Army Medical University, Chongqing, China

**Keywords:** prostate cancer, meta-analysis, pelvic floor prostatectomy, exercise, urinary incontinence

## Abstract

**Objective:**

We performed a systematic review and meta-analysis to evaluate the effect of preoperative pelvic floor muscle exercise on urinary incontinence after radical prostatectomy.

**Methods:**

We searched the literature for randomized controlled trials evaluating the diagnostic analysis of preoperative pelvic floor muscle exercise (PFME) and postprostatectomy incontinence in the MEDLINE, EMBASE, PubMed, Cochrane Central Register of Controlled Trials (CENTRAL), PsycINFO, China Biomedical Literature Database, China Journal Full-text Database, Wanfang Database and Weipu Database. The retrieval time limit is from the establishment of the database to January 2023. We used a risk ratio with accompanying 95% confidence interval (CI) to express estimates. Reviewer Manager (RevMan) 5.1.0 was used to complete all statistical analyses.

**Results:**

Twelve studies were included based on the selection criteria. The total number of patients included in the final analysis was 1,365. At 1th month, there was no difference in continence rates between the groups [odds ratio (OR): 0.47; 95% confidence interval (CI), 0.22–1.02, *p* = 0.06]. At 3th month, there was statistically significant difference in PFME group before operation (OR: 0.61; 95% CI, 0.37–0.98, *p* = 0.04). At 6th and 12th months, there was no difference between groups (OR: 0.57; 95% CI, 0.28–1.17, *p* = 0.13), (OR: 0.56; 95% CI, 0.27–1.15, *p* = 0.12).

**Conclusion:**

Preoperative pelvic floor muscle exercise can improve postoperative urinary incontinence at 3rd months after radical prostatectomy, but it cannot improve urinary incontinence at 6th months or longer after surgery, which indicates that preoperative PFME can improve early continence rate, but cannot improve long-term urinary incontinence continence rate.

## 1. Background

Prostate cancer is one of the most common malignant tumors in male genitourinary system. In Europe and the United States, the incidence rate of prostate cancer ranks first among male cancers, and the mortality rate ranks second (1, 2). Radical prostatectomy (RP) is the most common treatment for patients with localized prostate cancer. However, urinary incontinence after RP is a common complication. Most patients will have incontinence after removing the catheter. With the development of new surgical methods (3), despite better understanding of anatomy and improved surgical techniques, it is reported that the prevalence of urinary incontinence patients after prostate cancer surgery is still between 4 and 31% (4). The exact mechanism of urinary incontinence after radical prostatectomy is complex and unclear, but it is generally believed that it is mainly caused by sphincter injury and/or detrusor overactivity (5–8). Pelvic floor muscle exercise, as one of the conservative treatment methods for urinary incontinence after prostate cancer surgery, has been widely used in clinic (9–11), and many studies have investigated the effect of peri/postoperative PFME, with or without biofeedback, on the recovery of continence after RP, but results are conflicting. The aim of this systematic review and meta-analysis was to analyze published RCTs that these studies investigated the effect of preoperative pelvic floor muscle exercise on postoperative incontinence recovery, providing evidence for clinical nursing application.

## 2. Methods

This review was designed according to the framework recommended by the Cochrane Collaboration. We reported all results in accordance with the Preferred Reporting Items for Systematic Reviews and Meta-Analyses statement (12). Research Ethics Committee approval was not required for this study because our study was conducted based on published data.

### 2.1. Eligibility criteria

This systematic review included the use of pelvic floor muscle exercising in all studies. Eligible studies compared the effects of increased pelvic floor muscle exercising before operation and only postoperative pelvic floor muscle exercising on the recovery of postoperative urinary incontinence. No matter the publishing status, language or size.

Studies were excluded if they (a) were not RCTs, (b) did not indicated whether it is preoperative or postoperative, (c) were in a language for which a translation to English was not available or (d) were unpublished studies with only the abstracts presented at national and international meetings.

Included studies focused on men of all ages undergoing RP. To be eligible, in the intervention group had to involve a form of preoperative PFME with or without guidance and with or without biofeedback. Studies without controls (without preoperational PFME) and preoperational urinary incontinence were excluded. The main outcomes of interest were the incidence rate of urinary incontinence at 1th, 3th, 6th and 12th month after surgery.

### 2.2. Search methods for the identification of studies

We searched for studies on the MEDLINE, EMBASE, PubMed, Cochrane Central Register of Controlled Trials (CENTRAL), PsycINFO, China Biomedical Literature Database, China Journal Full-text Database, Wanfang Database and Weipu Database up to January 2023, without language restrictions. A controlled vocabulary was used (MeSH terms for MEDLINE and Cochrane; EMTREE for EMBASE). Keywords and their synonyms were used to sensitize the search, including “pelvic floor prostatectomy” or “Levator ani muscle” or “prostate cancer” and “exercise” or “RP” or “PFME” or “pelvic floor prostatectomy exercise” or “RP” and “PFME” or “pelvic floor prostatectomy training” or “PFMT.” The retrieval was conducted in the form of free words with a keyword.

For the identification of RCTs in PUBMED, the optimally sensitive strategy developed for the Cochrane Collaboration was used. For the identification of RCTs in EMBASE and other databases, a search strategy using similar terms was adopted. In the search strategy, there were four groups of keywords: study design, participants, interventions and outcome measures.

We analyzed the reference lists of all eligible articles to identify other potentially eligible studies. For ongoing studies or when any data were to be confirmed or additional information was needed, the authors were contacted by e-mail.

The previously described search strategy was used to obtain titles and abstracts of studies that were relevant for this review. Each identified abstract was independently evaluated by two authors. If at least one of the authors considered one reference eligible, the full text was obtained for complete assessment. Two reviewers independently assessed the full text of the selected articles to verify whether they met the criteria for inclusion or exclusion. In case of any disagreement, the authors discussed the reasons for their decisions, and a consensus was reached.

Two authors, independently blinded, extracted descriptive and outcome data from the included studies using a standardized form developed by the authors and adapted from the Cochrane Collaboration’s model for data extraction. We considered (a) aspects of the study population, such as the average age and sex; (b) aspects of the intervention performed (sample size, type of stabilization exercise performed, presence of supervision, frequency and duration of each session); (c) follow-up (if the patients included were analyzed); (d) loss to follow-up (if there was a loss in the sample); (e) outcome measures; and (f) presented results. Another author resolved disagreements. Any additional information required from the original author was requested by e-mail.

The quality of evidence was independently scored by two researchers based on the PEDro scale, which consisted of 11 items based on the Delphi list. One item on the PEDro scale (eligibility criteria) is related to external validity and is generally not used to calculate the method score, leaving a score range of 0–10.

### 2.3. Statistical assessment

The meta-analysis was performed with RevMan (Version 5.3. Copenhagen: The Nordic Cochrane Centre, The Cochrane Collaboration, 2014), and ORs and 95% CIs were used to summarize the outcomes. First, the heterogeneity of the research problem was assessed by the Cochran test. The significance level for the Cochran test was set at *α* = 0.1 as recommended by authors such as Sedgwick (13). We used Cochrane *Q* to qualitatively describe the heterogeneity across eligible studies, and then, the *I* ^2^ statistic was used to quantitatively estimate the heterogeneity. A low degree of heterogeneity was indicated by *p* ≥ 0.1 and *I* ^2^ ≤ 50%, and a high degree of heterogeneity was indicated by *p* < 0.1and *I* ^2^ > 50%. If the heterogeneity was high, we performed a further analysis of the heterogeneity sources. If there was no significant clinical heterogeneity, the random effects model was used for the meta-analysis. In this study, we performed all statistical analyses based on the random-effect model because heterogeneity cannot be omitted in reality.

### 2.4. Grade framework to rate the certainty of the evidence

The GRADE framework, a grading system for the quality of evidence, was used to classify the quality of evidence for outcome indicators. All RCTs were included in this study. RCT was set as the highest level of evidence. There are five factors that can reduce the quality of evidence: research limitations, publication bias, research inaccuracy, research inconsistency and indirectness of research results.

## 3. Results

### 3.1. Identification of literature

**Figure 1:**
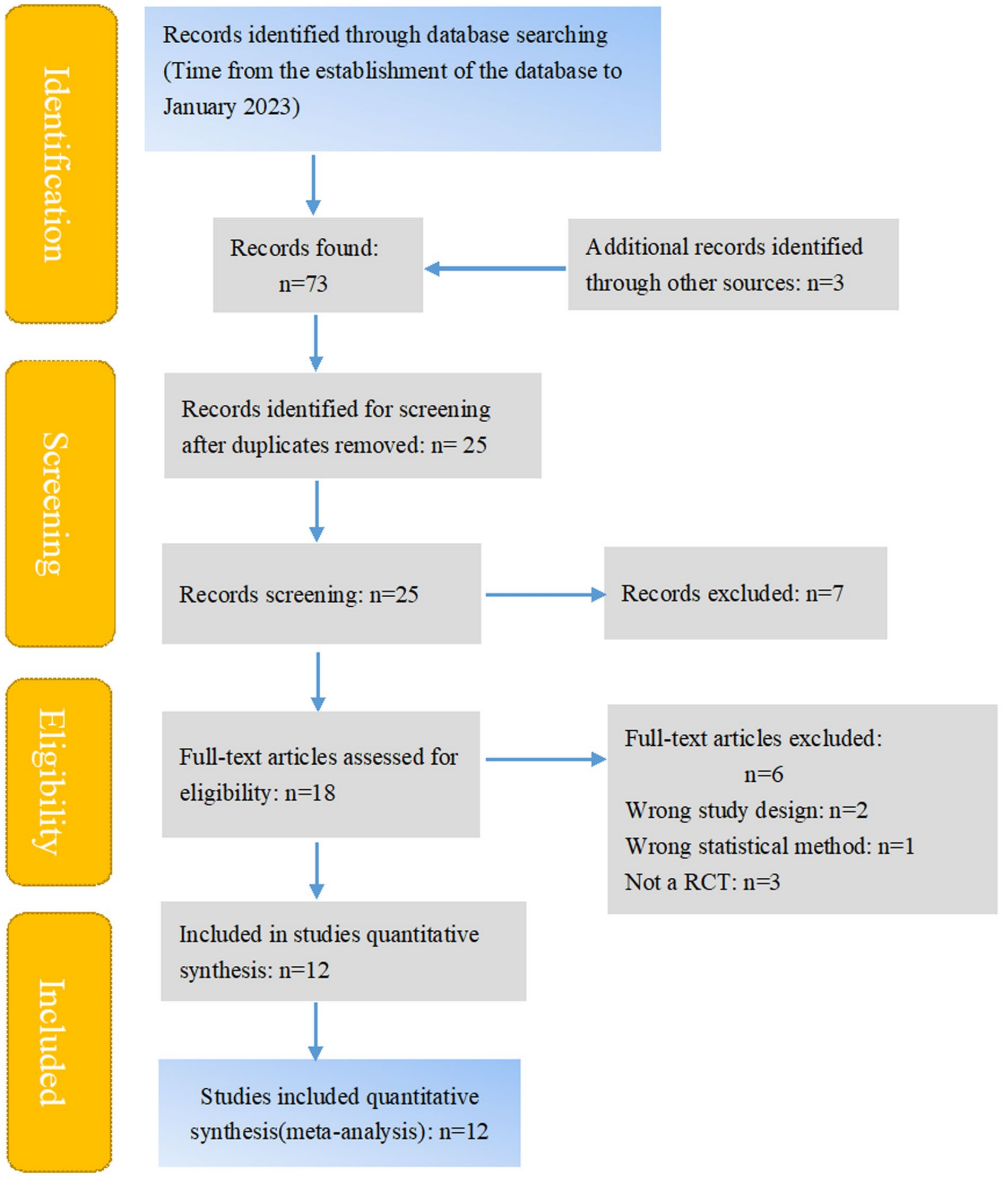
Search and selection of studies for systematic review according PRISMA.

The initial search led to the identification of 73 abstracts, from which 25 studies were considered potentially relevant and retrieved for detailed analysis. After a complete reading of 25 articles, 13 were excluded. Finally, 12 papers met the eligibility criteria. Figure 1 shows the PRISMA flow diagram of study selection for this review.

### 3.2. Characteristics of all included studies

The characteristics of the included studies are documented in Table 1. All eligible studies were published between 2000 and 2021. Among these studies, 3 were conducted in the United States, 3 in the Netherlands, 2 in Italy, 2 in China, and 1 in Australia and Spain. The sample size of each individual study ranged from 32 to 84, and the mean age ranged from 59 to 72.5 years. All studies were published in academic journals in full text. Eight studies reported that preoperative pelvic floor muscle exercise had effect or early effect, and four reported that exercise had no difference. There was a variety of different pelvic floor muscle exercising regimens with or without biofeedback. There were also differences in the amount of detail with which the regimens were described in each study. The exercise regimens are summarized in Table 1.

**Table 1:** Characteristics of trials included in the review.

Study	Country	mean age (exp vs. con)	Pelvic floor muscle training regimen	Length and timing of preoperative PFME	Length of follow-up, mo	Number (exp vs. con)	Training results	Incontinence (exp vs. con)			
								1mo	3mo	6mo	12mo
Bales 2000	United States	59.3 vs. 60.9	Guided, surface electrode	First session 2–4 wk. preoperatively 4times/d	≤6	47 vs. 50	N	38 vs. 38	20 vs. 19	3 vs. 2	_
Burgio 2006	United States	60.7 vs. 61.1	Visual biofeedback and rectal probes	1–3 sessions preoperatively 1time/d	≤6	57 vs 55	Y	49 vs. 51	_	3 vs. 11	_
Collado 2013	Spain	–	Surface electrode, incorporates abdominal hypopressive techniques， guided	First session 3 wk. preoperatively 1time/d	12	87 vs. 92	Y	_	60 vs. 64	35 vs. 43	12 vs. 13
Patel 2013	Australia	60 vs. 62	Guided, verbal and visual feedback	First session ≥4 wk. preoperatively	3	152 vs. 132	Y	_	41 vs. 50	_	_
Geraerts 2013	Netherlands	61.88 vs. 62.04	Guided, visual feedback, digital palpation and EMG rectal probes	First session3 wk. preoperatively	12	85 vs. 85	N	41 vs. 41	18 vs 14	5 vs. 5	2 vs. 4
Dijkstra-Eshuis2013	Netherlands	63.7	Guided, visual feedback, digital palpation and EMG rectal probes	First session 4 wk. preoperatively	12	65 vs. 56	N	_	_	_	38 vs. 45
Dubbelman 2010	Netherlands	64 vs. 64	Guided, verbal and visual feedback	First session 1 d preoperatively	≤6	34 vs. 36	Y	_	_	24 vs. 27	_
Centemero 2010	Italy	60.5 vs. 57.5	guided, verbal and visual feedback	First session 30d preoperatively	3	59 vs. 59	N	33 vs. 47	24 vs. 27	_	_
Tienforti 2012	Italy	64 vs. 67	Guided, verbal and visual feedback	First session 1 d preoperatively	≤6	16 vs. 16	Y	10 vs. 16	8 vs. 15	6 vs. 15	_
Parekh 2003	United States	61.6 vs. 55.5	Guided, visual feedback, digital palpation and EMG rectal probes	1–3 sessions preoperatively, not stated when	12	19 vs. 19	Y	_	6 vs. 12	_	_
Wu chengjie 2016	China	67.6 vs. 67.8	Bladder training	First session 2 wk. preoperatively	2	32 vs. 32	Y	2 vs. 12	_	_	_
Ding xixi 2021	China	60.24 vs. 60.35	Bladder training	First session1 wk. preoperatively	3	40 vs 40	Y	_	3 vs. 11	_	_

mo, month; exp, experimental group; con, control group; wk, week; EMG, electromyography; Y, yes; N, no.

### 3.3. Risk of bias

Each of the studies was scored using the PEDro scale (14). Table 2 presents the results of the individual assessments by the PEDro scale. The overall quality of all studies was fair to good, but the therapists and participants were not blinded in the design of all articles. All articles followed eligibility criteria and source of participants; random allocation; baseline adequate follow-up; between-group comparisons; and point estimates and variability. 4 articles lacked concealed allocation, 7 articles lacked blind assessors, and 3 articles lacked intention-to-treat analysis.

**Table 2:** Study quality on the PEDro scale.

	Study	1	2	3	4	5	6	7	8	9	10	11	Total
1	Bales 2000	√	√		√				√	√	√	√	6
2	Burgio 2006	√	√	√	√			√	√	√	√	√	8
3	Collado 2013	√	√	√	√				√		√	√	6
4	Patel 2013	√	√		√				√	√	√	√	6
5	Geraerts 2013	√	√	√	√	√	√	√	√	√	√	√	10
6	Dijkstra-Eshuis 2013	√	√	√	√	√	√	√	√	√	√	√	10
7	Dubbelman 2010	√	√	√	√	√		√	√	√	√	√	9
8	Centemero 2010	√	√	√	√	√	√		√	√	√	√	9
9	Tienforti 2012	√	√		√	√			√	√	√	√	7
10	Parekh 2003	√	√		√	√		√	√	√	√	√	8
11	Wu chengjie 2016	√	√	√	√				√		√	√	6
12	Ding xixi 2021	√	√	√	√				√		√	√	6

1, eligibility criteria and source of participants; 2, random allocation; 3, concealed allocation; 4, baseline comparability; 5, blinded participants; 6, blinded therapists; 7, blind assessors; 8, adequate follow- up; 9, intention- to- treat analysis; 10, between- group comparisons; 11, point estimates and variability. Item 1 does not contribute to the total score.

### 3.4. Continence outcomes

**Figure 2:**
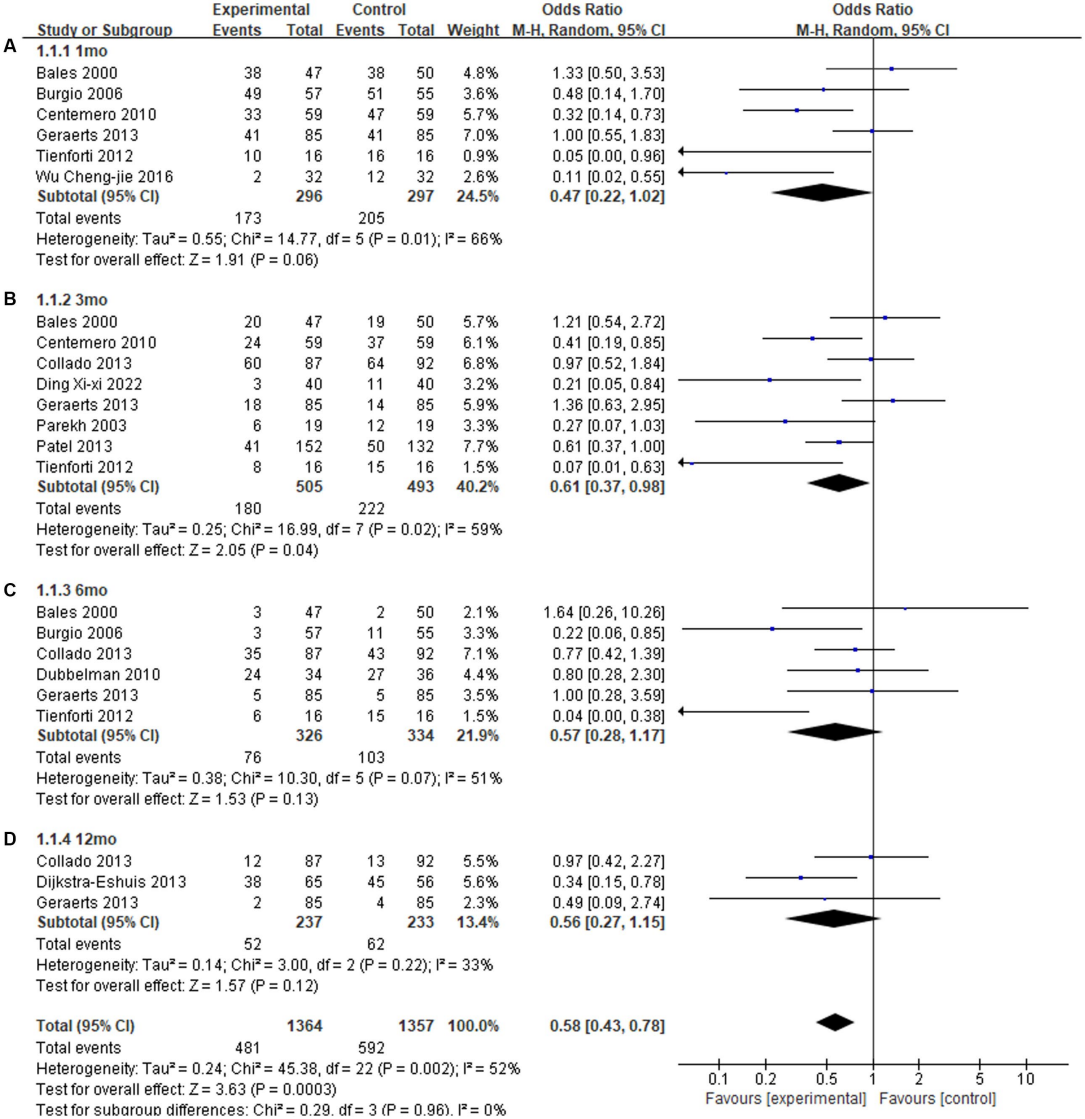
Forest plot of odds ratios for urinary incontinence with preoperative pelvic floor muscle exercise (PFME) compared with no preoperative PFME at **(A)** 1 mo, **(B)** 3 mo, **(C)** 6 mo and **(D)** 12 mo following radical prostatectomy. CI, confidence interval; M-H, Mantel–Haenszel.

**Figure 3:**
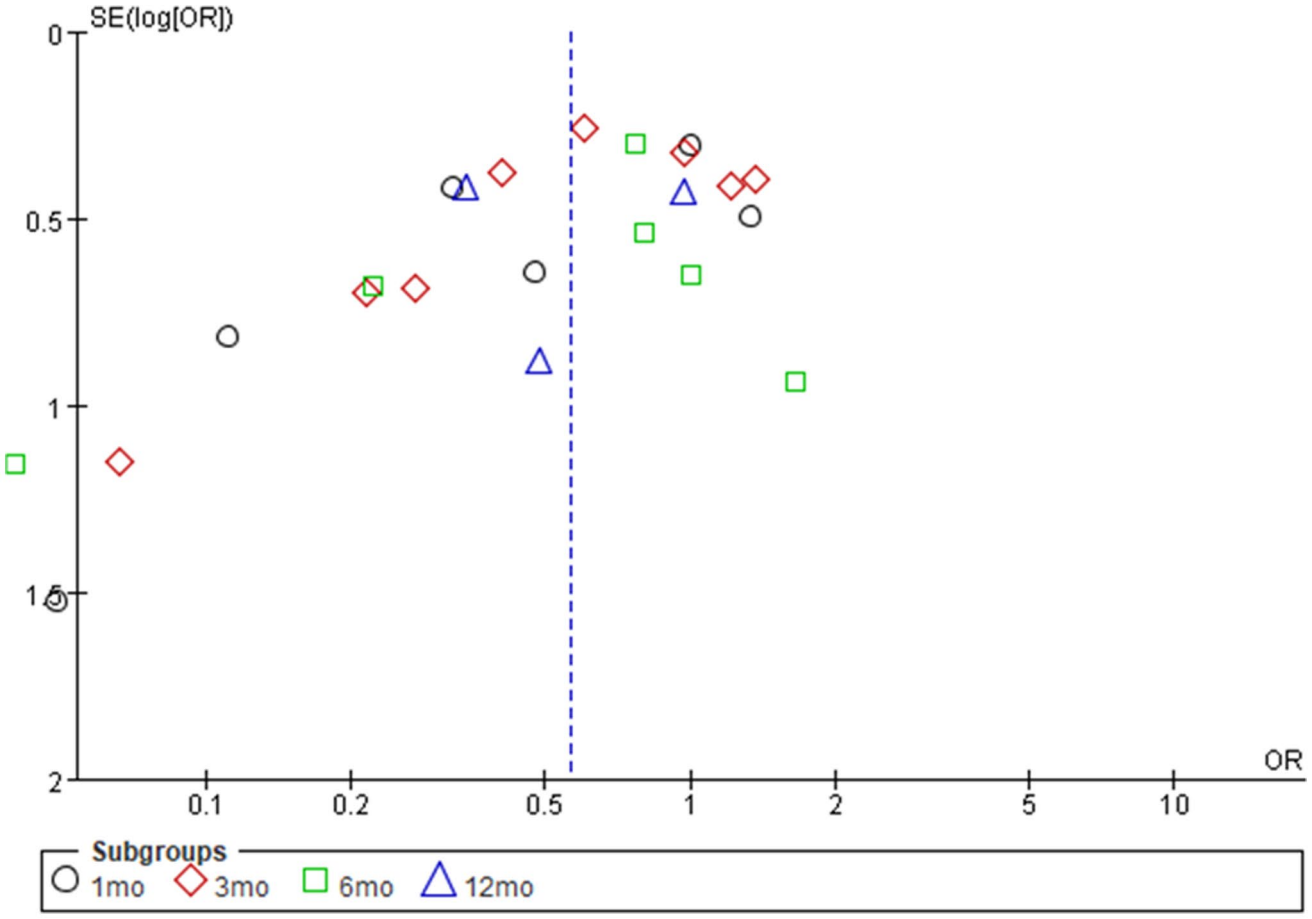
Funnel diagram of odds ratios for urinary incontinence with preoperative pelvic floor muscle exercise (PFME) compared with no preoperative PFME.

In this meta-analysis, we found that there were significantly lower rates of postoperative incontinence at 3th month in the preoperative PFME group compared with the control group, with an OR of being incontinent of 0.61 (*p* = 0.04). There was no significant difference in postoperative incontinence rates at 1th month (OR: 0.47; *p* = 0.06), 6th month (OR: 0.57; *p* = 0.13) or 12th month (OR: 0.56; *p* = 0.12), the overall effect (OR: 0.58; *p* = 0.0003) (Figure 2). The funnel diagram is basically symmetrical, as shown in Figure 3. It is less likely to consider the existence of publication bias.

## 4. Discussion

Our research shows that preoperative pelvic floor muscle exercise is beneficial to the early recovery of urinary incontinence after RP, but it has no significant effect on the recovery of urinary incontinence in the medium and long term.

### 4.1. The role of pelvic floor muscle exercise in the recovery of urinary incontinence

Urinary incontinence after radical resection of prostate cancer is mainly caused by the injury of the urethral sphincter during the operation. According to the latest development, the concept of sphincter relaxation caused by the inherent sphincter defect after the operation is considered to be the cause of the disorder of the male overall system, which often causes physical and psychological pain of patients, increases the psychological burden of patients, and significantly reduces the quality of life (8).

The European Urological Society recommends pelvic floor muscle exercise as the main treatment for postoperative urinary incontinence recovery. Pelvic floor muscle exercise is a simple and easy method to promote recovery. The purpose of pelvic floor muscle exercise is to continuously strengthen the contraction of the levator ani muscle to increase the tension of the urethral fascia, maintain a good tension of the distal urethral sphincter, keep the pressure of the urethra higher than the internal pressure of the bladder, and improve the ability to control urination. At the same time, this exercise can effectively improve the pelvic floor nerve function of patients, enhance the muscle contractility and tension, and increase the volume of urethral sphincter (15, 16). However, according to the research published so far, the role of preoperative PFME with or without biofeedback is still controversial. Burgio et al. 8 studies showed that increasing preoperative PFME was beneficial to the recovery of urinary incontinence after RP (17–24), while Bales et al. 4 studies showed that there was no significant difference between increasing preoperative PFME and recovering urinary incontinence after RP (25–28). We analyzed the reasons for this difference, which may be as follows: firstly, the technical level of the surgeon and the choice of surgical method have a certain impact on postoperative recovery. Secondly, the time of starting PFME before operation is different, the earliest 1 month before operation and the latest 1 day before operation; There are also those that only mention preoperative, but no indication of time. Thirdly, the number of preoperative exercises is different. Most studies only mention the start time, but not the number of times. Among them, the doctor suggested four times a day, but the study did not indicate whether the patient completed the exercise as recommended by the doctor. Fourthly, the feedback methods are inconsistent, including visual, oral, probe and surface electrode. Evaluators may have deviations due to different knowledge level structures. Fifthly, there is a deviation in the definition of urinary incontinence. The same result may be the opposite in different studies. Finally, pelvic floor muscle exercise needs patients to be able to cooperate and persist. Because of the large age deviation of prostate cancer patients, although physical therapists or nurses guide them to exercise, due to the complexity of pelvic floor muscle anatomy, it is difficult for patients to determine which muscles are contracting and whether the contraction is correct; At the same time, there may be insufficient PFME frequency and intensity, which will affect the final result. So it is difficult to judge whether exercise is effective (29, 30). Therefore, it is suggested that the follow-up related research should provide scientific basis for clinical practice through standardized feedback mode, professional guidance of medical staff and long-term follow-up on the impact of preoperative PFME on urinary incontinence after RP.

### 4.2. Limitations of this study

This study is limited by the number of studies available for analysis, Eastern and Western populations, age span, training methods, cycles, intensity, effectiveness, follow-up time, and limited number of patients. The resources used in this meta-analysis are all from the literature, so it is impossible to obtain more detailed information. Because the literature is limited by objective factors such as source, amount of information provided, different PFME protocols, definitions of urinary incontinence, with or without biofeedback and control of confounding factors, it may affect the conclusion of this study to some extent. Therefore, it is necessary to carry out prospective research focusing on the control of confounding factors while evaluating the problem more scientifically and comprehensively. In addition, in clinical trials, there are many uncontrollable factors, which make it difficult to achieve randomization, concealment and blinding of allocation schemes; Therefore, the analysis results in this paper are for reference only.

## 5. Conclusion

In conclusion, our research shows that preoperative PFME may aid early urinary incontinence recovery of patients after RP. However, due to limited data, it is impossible to draw a clear conclusion on this issue. In the future, it is necessary to carry out a multicenter, large-sample follow-up study to further explore and confirm the role of PFME in the recovery of urinary incontinence after RP.

## Author contributions

JZheng and YC: study design. LZh and YC: conceptualization. LZh: statistics. JZheng: review and editing. All authors have read and agreed on the published version of the manuscript.

## Funding

This study was supported by the Key Project for Clinical Innovation of Army Medical University (CX2019LC107) and the Joint Project of Chongqing Municipal Health Commission and Science and Technology Commission (2023GDRC007).

## Conflict of interest

The authors declare that the research was conducted in the absence of any commercial or financial relationships that could be construed as a potential conflict of interest.

## Publisher’s note

All claims expressed in this article are solely those of the authors and do not necessarily represent those of their affiliated organizations, or those of the publisher, the editors and the reviewers. Any product that may be evaluated in this article, or claim that may be made by its manufacturer, is not guaranteed or endorsed by the publisher.
